# Adipocyte mitochondrial genes and the forkhead factor FOXC2 are decreased in type 2 diabetes patients and normalized in response to rosiglitazone

**DOI:** 10.1186/1758-5996-3-32

**Published:** 2011-11-18

**Authors:** Joakim Håkansson, Björn Eliasson, Ulf Smith, Sven Enerbäck

**Affiliations:** 1 Department of Medical and Clinical Genetics, Institute of Biomedicine University of Gothenburg, Göteborg, Sweden; 2 PharmaSurgics AB, Arvid Wallgrens backe 20, Göteborg, Sweden; 3 The Lundberg Laboratory for Diabetes Research, Department of Molecular and Clinical Medicine, The Sahlgrenska Academy at the University of Gothenburg, Göteborg, Sweden

## Abstract

**Background:**

FOXC2 has lately been implicated in diabetes and obesity as well as mitochondrial function and biogenesis and also as a regulator of mtTFA/Tfam. In this study, the expression of FOXC2 and selected genes involved in mitochondrial function and biogenesis in healthy subjects and in a matched cohort with type 2 diabetes patients before and after treatment with rosiglitazone was determined. Quantitative real time PCR was used to analyze both RNA and DNA from biopsies from subcutaneous adipose tissue.

**Methods:**

Blood samples and subcutaneous abdominal fat biopsies were collected from 12 T2D patients, of which 11 concluded the study, pre-treatment and 90 days after initiation of rosiglitazone treatment, and from 19 healthy control subjects on the first and only visit from healthy subjects. Clinical parameters were measured on the blood samples. RNA and DNA were prepared from the fat biopsies and gene expression was measured with real time PCR.

**Results:**

The expression level of genes in the mitochondrial respiratory complexes I - IV were significantly downregulated in the diabetic patients and restored in response to rosiglitazone treatment. Rosiglitazone treatment also increased the relative number of mitochondria in diabetic patients compared with controls. Furthermore, the transcription factors FOXC2 and mtTFA/Tfam displayed a response pattern identical to the mitochondrial genes.

**Conclusions:**

FOXC2, mtTFA/Tfam and subunits of the respiratory complexes I - IV show equivalent regulation in gene expression levels in response to TZD treatment. This, together with the knowledge that FOXC2 has a regulatory function of mtTFA/Tfam and mitochondrial biogenesis, suggests that FOXC2 has a possible functional role in the TZD activated mitochondrial response.

## Background

Insulin resistance is strongly associated with obesity and a principal underlying defect in type 2 diabetes (T2D). Insulin resistance also underpins the abnormalities associated with the metabolic syndrome. Thiazolidinediones (TZDs) are peroxisomal proliferator-activated receptor (PPAR) - gamma agonists acting as powerful insulin sensitizers with glucose lowering effects similar to sulphonylureas and metformin. Apart from this, TZDs have also been linked to a plethora of other effects such as improved pro-coagulant state, blood pressure lowering effects and anti-inflammatory activity [1,2]. While some TZDs have been associated with impaired mitochondrial function [3] others have shown induction of uncoupling protein-1 in white adipose tissue depots, which would be compatible with enhanced mitochondrial activity [4].

The aim of this study was to analyze TZD mediated effects on markers of mitochondrial function in adipose tissue. We examined biopsies from healthy controls and a matched cohort of patients with type 2 diabetes prior to and during treatment with rosiglitazone. Clinical variables such as blood pressure, fat mass and body mass index (BMI) were compared as well as blood lipid and insulin/glucose levels. From adipose tissue biopsies we prepared DNA and total RNA, which enabled us to compare the relative density of mitochondria as well as expression levels of genes of the mitochondrial respiratory chain complexes I - IV, including NDUFS4, SDHA, cytochrome b and COX II, and their responses to rosiglitazone treatment. We also analyzed the expression of the nuclear-encoded mitochondrial transcription factor A (mtTFA also referred to as Tfam), crucial in mitochondrial transcription and biogenesis [5-8], and FOXC2, which lately has been implicated both as a promoter of brown fat development and insulin sensitivity [9-11], an important factor in mitochondrial function and biogenesis [12] as well as a regulator of mtTFA/Tfam expression [12].

## Results

### Clinical characteristics of the subjects and treatment effects

**Figure 1:**
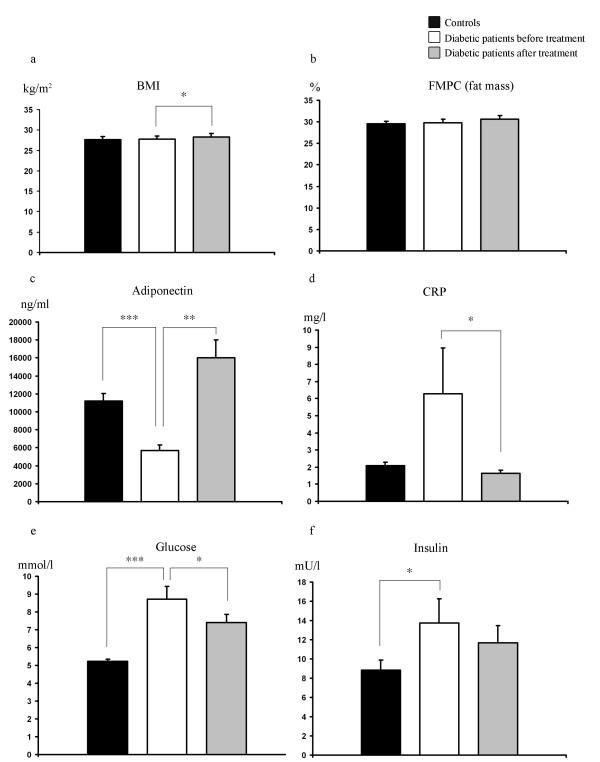
**Clinical characterization and treatment effects**. The clinical variables BMI (a), FMPC (b),adiponectin (c), CRP (d), glucose (e) and insulin (f) of the healthy controls and diabetic patients before and after treatment with rosiglitazone for 90 days. Bars represent mean and error bars SEM. * = p < 0.05, ** = p < 0.01, *** = p < 0.001.

There were no statistically significant differences between the T2D patients at baseline and the control subjects in BMI, fat mass and blood pressure. As expected, fasting plasma glucose, serum insulin, HbA1c, HOMA-IR as well as HS-CRP were clearly elevated in the T2D patients, while adiponectin was suppressed. There was also a tendency towards typical blood lipid abnormalities in the T2D patients (low HDL, high free fatty acids and triglycerides) but only the small reduction in total cholesterol and HDL were statistically significant when compared with the control subjects (Figures 1 and Additional file 1). Rosiglitazone treatment led to a small increase in BMI and total cholesterol, as well as marked increases in adiponectin and serum triglycerides, but reductions in plasma glucose, HOMA-IR, HS-CRP, and systolic blood pressure (Figure 1 and Additional file 1 and 2).

### Mitochondrial number and steady state levels of mRNA encoding mitochondrial respiratory chain proteins

**Figure 2:**
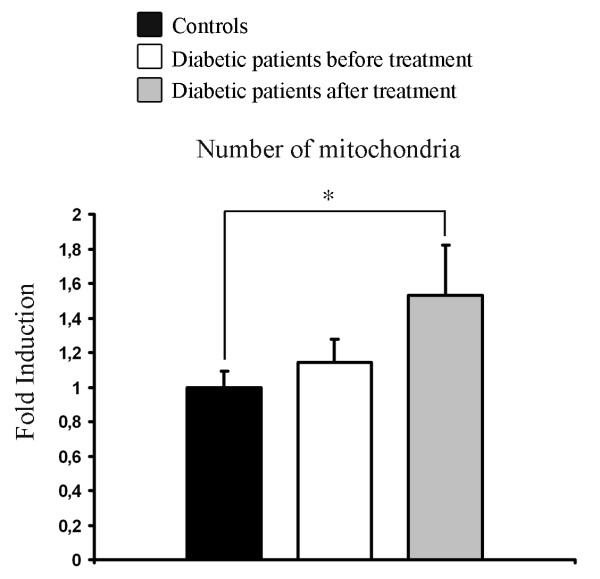
**Number of mitochondria in controls and patients with type II diabetes**. Real-time PCR quantification of the number of mitochondria based on quantification of the mitochondria encoded gene cytochrome b and the nuclear encoded genes cyclophilin A and NRF-1. Bars represent mean and error bars SEM. * = p < 0.05.

To quantify and compare the relative numbers of mitochondria in the groups examined we set up a qrtPCR assay to normalize the number of copies of the mitochondria encoded gene cytochrome b against the nuclear encoded genes cyclophilin A and NRF1. This ratio of genomic DNA derived form the nucleus and the mitochondrion reflects the relative abundance of mitochondria in the collected tissue samples. While there was no significant difference in this ratio when comparing controls with T2D patients, there was a significant increase in relative abundance of mitochondria as a consequence of rosiglitazone treatment (Figure 2).

**Figure 3:**
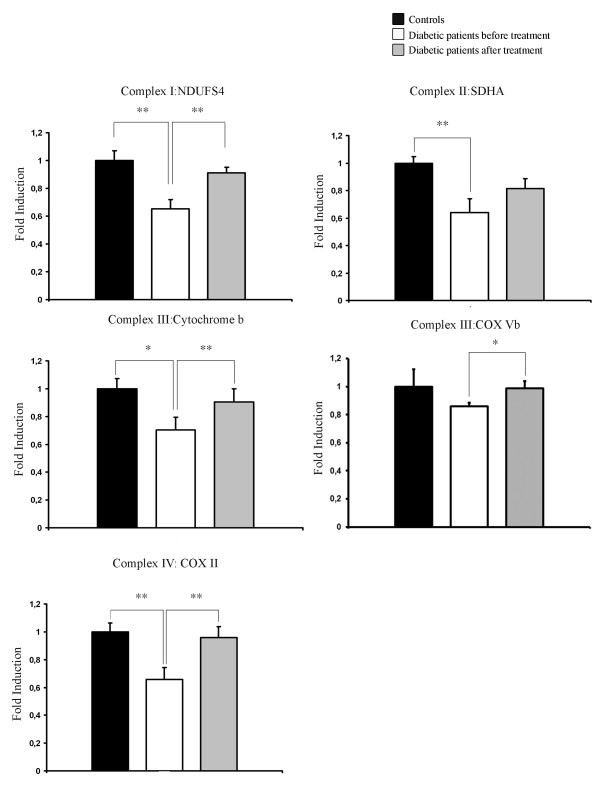
**mRNA levels of genes coding for subunits in the electron transport chain**. Relative expression levels of genes coding for subunits of the electron transport chain in healthy controls and diabetic patients before and after treatment with rosiglitazone for 90 days. (a) NDUFS4 in complex I, (b) SDHA in complex II, (c) cytochrome b in complex III, (d) COX Vb in complex III, (e) COX II in complex IV. Bars represents mean and error bars SEM. * = p < 0.05, ** = p < 0.01.

We investigated mRNA steady state levels of mitochondrial genes encoding proteins in the respiratory chain. Levels of mRNA encoding members of complex I (NDUFS4), II (SDHA), III (Cytochrome b) and IV (COX II) were measured using real time PCR. As can be deduced from Figure 3, there was a significant down regulation of these genes in adipose tissue of patients with T2D. In response to treatment this reduction is significantly reversed for all genes measured except SDHA of complex II. Furthermore, there was a significant correlation between the expression of the mitochondrial genes NDUFS4, Cytochome b, COX II and mtTFA/Tfam and the levels of adiponectin, insulin and glucose. The gene expression level of SDHA significantly correlated with the glucose and insulin, but not adiponectin levels (data not shown).

**Figure 4:**
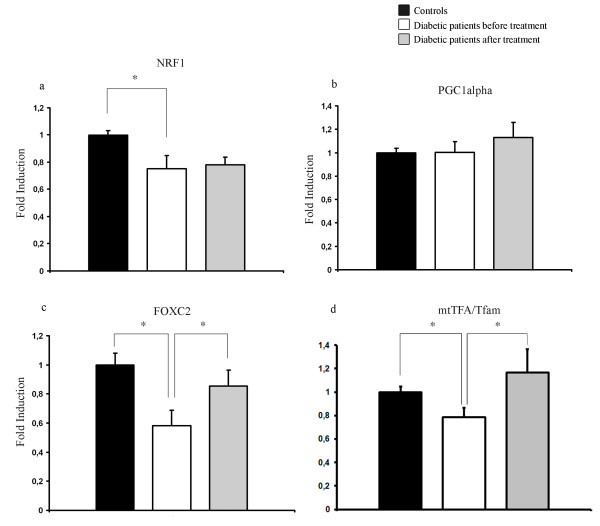
**mRNA levels of NRF1, FOXC2, PGC1α and mtTFA/Tfam**. Relative mRNA expression levels of NRF1 (a), PGC1α (b), FOXC2 (c) and mtTFA/Tfam (d) in healthy controls and diabetic patients before and after treatment with rosiglitazone for 90 days. Bars represent mean and error bars SEM. * = p < 0.05.

We also investigated mRNA levels for NRF1, PGC1α, mtTFA/Tfam as well as FOXC2, a forkhead family transcription factor, previously shown to increase the amount of mitochondria rich brown adipose tissue [9,11], and to stimulate mitochondrial function and biogenesis [12]. There was a significant decrease in mRNA levels of NRF1 when control subjects and T2D patients were compared (Figure 4), but there was no regulation in response to treatment. In the case of PGC1α no significant regulation was seen. On the other hand, FOXC2 and mtTFA/Tfam levels were significantly down regulated in the T2D cohort and significantly up regulated in response to treatment, thus mimicking the expression profiles of respiratory chain subunits (Figure 4).

## Discussion

Thiazolidinediones improve insulin resistance by enhancing insulin action in skeletal muscle, liver and adipose tissue through a mechanism that is not yet fully understood. Whereas biochemically TZDs are ligands for PPARγ and previous studies suggest that there is a correlation between anti-diabetic effect and PPARγ activation, some studies imply that also receptor independent TZD effects might contribute, as reviewed by Feinstein et al. [3]. In this study, we addressed the question of TZD related effects on mitochondrial function in human adipose tissue biopsies. Three groups were compared: healthy control subjects and a group of matched patients with non-symptomatic T2D on stable treatment with diet and/or hypoglycemic agents (not TZD) prior to and after treatment with rosiglitazone for 90 days.

While an increase in BMI was noted during treatment no increase in fat mass was observed (Figure 1). This is most likely a consequence of peripheral fluid retention, a well-known side effect of TZD treatment [13]. Systolic but not diastolic blood pressure was lowered in the treated group, a finding that agrees well with other studies that implicate a more pronounced lowering-effect on systolic pressure as compared with diastolic [14]. Also, the well-documented anti-inflammatory effect of TZD treatment, which may be in part mediated by increased adiponectin levels and visualized as a reduction in levels of C-reactive protein (Figure 1), can promote anti-atherosclerotic effects [15]. It cannot be ruled out that other factors, like physical activity, could contribute to the changes in the rosiglitazone-treated group, but the patients did not report changes in their lifestyles when asked.

Using a PCR based method we measured levels of mtDNA as well as nuclear DNA as described under "material and methods". This enabled us to calculate the ratio of mtDNA normalized against nuclear DNA reflecting the relative number of mitochondria. There was a significant increase in the relative amount of mtDNA when controls and treated patients were compared (Figure 4). This agrees well with earlier studies demonstrating that in response to pioglitazone patients with T2D increase levels of mtDNA in subcutaneous adipose tissue [16,17]. In the present study we extend what is known about TZD induced effects on mitochondrial function by not only measuring relative mtDNA levels but also steady state levels of mitochondria encoded subunits of respiratory complexes. In Figure 4 we demonstrate that subunits derived from complex I, II, III and IV are all significantly down regulated in patients with T2D. After rosiglitazone treatment, expression levels of these subunits were normalized or near normalized. This extends previous findings in which genes involved in oxidative metabolism have been shown to be positively regulated by TZDs [16]. Here we demonstrate that the mitochondrial genome in human adipose tissue, directly or indirectly, responds to rosiglitazone treatment as judged by regulation of respiratory chain subunits.

Another interesting finding is the co-variation of adiponectin levels with the profile of respiratory chain complexes. This has actually been suggested based on experiments in mice and using cell lines [18]. Thus, a possible regulatory role involving the mitochondria in regulation of adiponectin levels cannot be excluded, and by the experiments presented here this notion can be extended to include human adipose tissue. PGC1α has been shown to be positively regulated by TZDs and suppressed during insulin resistance [16,19]. In this study, we see a trend towards increased levels in response to rosiglitazone treatment but no difference between control and untreated T2D subjects. While in our material we see a significant down regulation of NRF1 in T2D patients, as has previously been reported [20], there is no significant regulation of PGC1α (Figure 4). This is in contrast to studies showing that PGC1α is positively regulated by TZDs and suppressed during insulin resistance [16,21]. The lack of TZD mediated regulation of NRF1 in adipose tissue gains support from a study by Bogacka et al. [16].

We also demonstrate that the transcription factor FOXC2 is subjected to significant regulation. This is interesting since FOXC2 has been implicated as a regulator of the amount of brown fat in rodents and also to increase the number of mitochondria and the mitochondrial function in response to its up-regulation [9,10,12]. Previously, we have shown reduced levels of FOXC2 in adipose tissue of insulin resistant subjects [11] and here we extend this observation to also include TZD-mediated regulation. Thus, FOXC2, previously linked to increased mitochondrial numbers in mice [9], is downregulated in patients with T2D and upregulated in response to TZD treatment. The profile of FOXC2 regulation, as depicted in Figure 4, matches that of adiponectin and those of the respiratory chain complex subunits.

Furthermore, we recently showed that FOXC2 has a regulatory function of mtTFA/Tfam [12], crucial for mitochondrial transcription and biogenesis [5-8]. In the present study we show the same transcriptional regulation of FOXC2 and mtTFA/Tfam in diabetic patients compared to healthy controls and in response to rosiglitazone treatment. Together, this not only establishes FOXC2 as a TZD responsive gene but it also suggests that FOXC2, in support of other evidence [12], plays a role in mitochondrial gene regulation in human adipose tissue.

In conclusion, the equivalent regulation in gene expression of FOXC2, mtTFA/Tfam and subunits of the respiratory complexes I - IV, in response to TZD treatment, together with the knowledge that FOXC2 has a regulatory function of mtTFA/Tfam and mitochondrial biogenesis, suggests a possible functional role for FOXC2 in the TZD activated mitochondrial response.

## Materials and methods

The regional ethical review board approved this explorative open trial, which was carried out in accordance with the principles of the Declaration of Helsinki. Informed consent was obtained from all patients.

### Subjects and treatment

We recruited 12 T2D patients (including 11 men and one woman) and 19 healthy control subjects (including 16 men and three post-menopausal women) via a newspaper advertisement. Patients on treatment with diet or oral hypoglycemic agents participated, and rosiglitazone 8 mg QD was added to the treatment regimen in accordance with the approved label. Before the study, three patients were on diet treatment, three on sulfonylurea, two on repaglinide, two on metformin and one on sulfonylurea and metformin combined.

Clinically significant diseases or symptoms, such as ischemic heart disease and thyroid disease as well as HbA1c > 10% were exclusion criteria. Seven patients were treated with antihypertensive agents and four with statins against hyperlipidemia. All patients were non-smokers but one subject used moist snuff. The patients were encouraged not to change their lifestyle habits during the study. One lean untreated patient (BMI 20) had previously been diagnosed as type 2 diabetic on clinical grounds but was excluded due to low serum insulin and C-peptide levels as well as rapidly deteriorating glycemic control. Thus, the results are based on the 11 patients who concluded the 90 days treatment period.

The healthy control subjects, who were individually matched to the patients with diabetes according to age and BMI, were all non-smokers but one subject used moist snuff. One subject was treated with an antihypertensive agent and one with a statin for hyperlipidemia.

### Clinical procedures

Blood samples and abdominal fat biopsies were collected pre-treatment and 90 days after initiation of rosiglitazone treatment from the diabetic patients, and on the first and only visit from healthy subjects. All blood sampling and examinations were done in the morning after an overnight fast and before the participants had taken any of their daily medication. The biopsies (approximately 300 mg) were taken from the subcutaneous adipose tissue in the paraumbilical region after local infiltrative anesthesia with lidocaine (10 mL, 0.5%). Plasma glucose, serum insulin and blood lipids were analyzed using standard laboratory methods. HbA1c was determined using high-performance liquid chromatography (Mono-S method). In this study, all HbA1c values were converted to NGSP standard levels using the formula: HbA1c (NGSP) = (0.923 × HbA1c (Mono-S) + 1.345 (R^2 ^= 0.998)[22]. High-sensitive C-reactive protein (HS-CRP) was analyzed with an immunoturbidimetric method. Total adiponectin was measured using the Human Adiponectin ELISA Kit (B-Bridge International, BioCat, GmbH, Germany). The proportion of body fat was determined using bioelectrical impedance (single frequency, 50 kHz; Animeter, HTS, Odense, Denmark). Blood pressure was measured in the supine position as a mean (mmHg) of two readings (Korotkoff 1 - 5), using a cuff of appropriate size.

### RNA and DNA isolation and cDNA synthesis

Total RNA was isolated from approximately 200 mg fat tissue with the RNeasy Lipid Tissue Mini kit (Qiagen) and treated with RNase free DNase (Qiagen) according to the manufacturer's protocol. The RNA concentration was measured on a Nanodrop ND-1000 (Saveen Werner). DNA was isolated from approximately 100 mg fat tissue using the Wizard Genomic DNA purification kit (Promega) according to the manufacturer's protocol. The 1st Strand cDNA Synthesis kit for RT-PCR (AMV) (Roche) was used for reverse transcription of 500 ng total RNA using a mix of random primer p(dN)_6 _and oligo dT_15_.

### Quantitative real time PCR

All real-time PCR assays contained 13 μl SYBR green PCR Master Mix (2×) (Applied Biosystems), 9 μl H_2_O, 1.5 μl of each forward and reverse primer (20 μM) and 1 μl cDNA or genomic DNA, except for FOXC2 for which the following composition was used: 13 μl TaqMan 2× Universal Master Mix (Applied Biosystems), 9.95 μl H_2_O, 0.65 μl forward primer (13 μM), 0.9 μl reverse primer (13 μM), 0.5 μl probe (5 μM) and 1 μl cDNA. The real-time PCR was performed with an ABI PRISM 7900HT (Applied Biosystems) with the following incubations: 50°C for 2 minutes, 95°C for 10 minutes and then 40 cycles of 95°C for 15 seconds and 60°C for 1 minute. Expected PCR products were confirmed on agarose gel electrophoresis (2%) and melting curve analysis. Real-time PCR was run on genomic and mitochondrial DNA to quantify the number of genomic copies, and on cDNA made from mRNA to quantify the number of transcripts. The real-time PCR primers were designed using Primer3 http://frodo.wi.mit.edu/primer3/.

The following primers were used on genomic DNA: Cytochrome b fwd 5'-tatccgccatcccatacatt-3' rev: 5'-ggtgattcctagggggttgt-3', Cyclophilin A fwd 5'-ttcatctgcactgccaagac-3' rev 5'-tcgagttgtccacagtcagc3', NRF1 fwd 5'-gtccagatccctgtgagcat-3'rev 5'-caatgtcaccacctccacag-3. Primers used on cDNA were: Cytochrome b fwd 5'-caacatctccgcatgatgaaa-3' rev 5'-ccataatttacgtctcgagtgatgtg-3', Cyclophilin A fwd 5'-catctgcactgccaagactga-3'rev 5'-ttcatgccttctttcactttgc-3', NRF1 fwd 5'-cctccaaacctaaccctgtcttta-3'rev 5'-cttccaggatcatgctcttgtactt-3', β-actin fwd 5'-gagctacgagctgcctgacg-3'rev 5'-gtagtttcgtggatgccacag-3, PGC1α fwd 5'-gccaaaccaacaactttatctcttc-3' rev 5'-cacacttaaggtgcgttcaatagtc-3', NDUFS4 fwd 5'-tccaagaaaagtttgcacca-3' rev 5'-cgcaataacatgcagtctgg-3', COX II fwd 5'-tgaagcccccattcgtataa-3' rev 5'-atgggcatgaaactgtggtt-3', COX Vb fwd 5'- actgggttggagagggagat-3' rev 5'-agacgacgctggtattgtcc-3', SDHA fwd 5'-tgggaacaagagggcatctg-3' rev 5'- ccaccactgcatcaaattcatg-3', FOXC2 fwd 5'-gcccagcagcaaactttcc-3'rev 5'-cccgagggtcgagttctca-3' probe 5'-tgttcaactcccaccggctggg-3', mtTFA/Tfam fwd 5'- gggttccagttgtgattgct-3' rev 5'- gccaagacagatgaaaacca-3'.

### Calculations and statistics

In a PCR assay, the relative number of mitochondria was determined by measuring mtDNA as the level of the mitochondrial gene cytochrome b and normalizing it, by geometric averaging, against nuclear DNA measured as the level of cyclophilin A and NRF-1. Similar methodology has been used by others [16]. To quantify the number of transcripts, the mRNA levels were normalized against cyclophilin A and β-actin by geometric averaging. All PCR reactions were run in triplicates and the mean C_t_-values were used. For statistical calculations Mann-Whitney U-test, Wilcoxon's matched pairs signed rank sum test and Student's t-test were used as appropriate. The correlation was analyzed on all subjects, according to Spearman.

## Competing interests

The authors declare that they have no competing interests.

## Authors' contributions

SE, US and BE participated in the planning and designing of the study and writing the manuscript. BE collected the clinical samples and performed the clinical tests. JH prepared the clinical samples, performed the real-time PCR analysis and participated in writing the manuscript. All authors have read and approved the final manuscript.

## Supplementary Material

Additional file 1 **Clinical variables I**. The clinical variables systolic pressure (a), diastolic pressure (b), the calculated value for HOMA-IR (c) and Hba1c (d) of the healthy controls and diabetic patients before and after treatment with rosiglitazone for 90 days. Bars represent mean and error bars SEM. * = p < 0.05, ** = p < 0.01, *** = p < 0.001. Click here for file

Additional file 2 **Clinical variables II**. The clinical variables cholesterol (a), LDL (b), HDL (c), free fatty acids (d) and triglycerides (e) of the healthy controls and diabetic patients before and after treatment with rosiglitazone for 90 days. Bars represent mean and error bars SEM. * = p < 0.05. Click here for file
