# Supplementary figures and images for: Adipocyte mitochondrial genes and the forkhead factor FOXC2 are decreased in type 2 diabetes patients and normalized in response to rosiglitazone
Source: Diabetol Metab Syndr. 2011 Nov 18;3:32. doi: 10.1186/1758-5996-3-32 (PMC3230127; doi:10.1186/1758-5996-3-32)

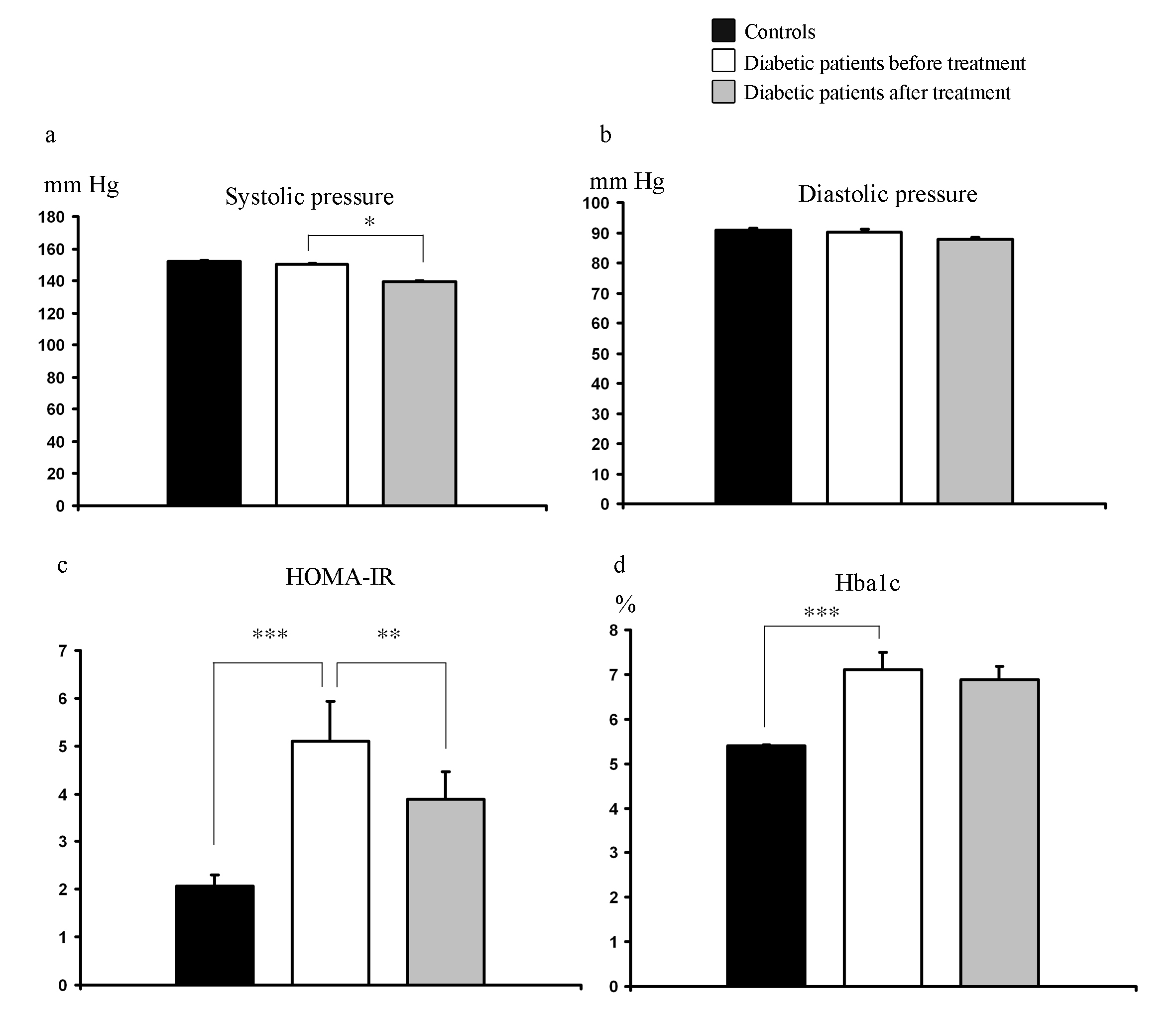

Supplement: Additional file 1 — Clinical variables I. The clinical variables systolic pressure (a), diastolic pressure (b), the calculated value for HOMA-IR (c) and Hba1c (d) of the healthy controls and diabetic patients before and after treatment with rosiglitazone for 90 days. Bars represent mean and error bars SEM. * = p < 0.05, ** = p < 0.01, *** = p < 0.001. [file 1758-5996-3-32-S1.JPEG]

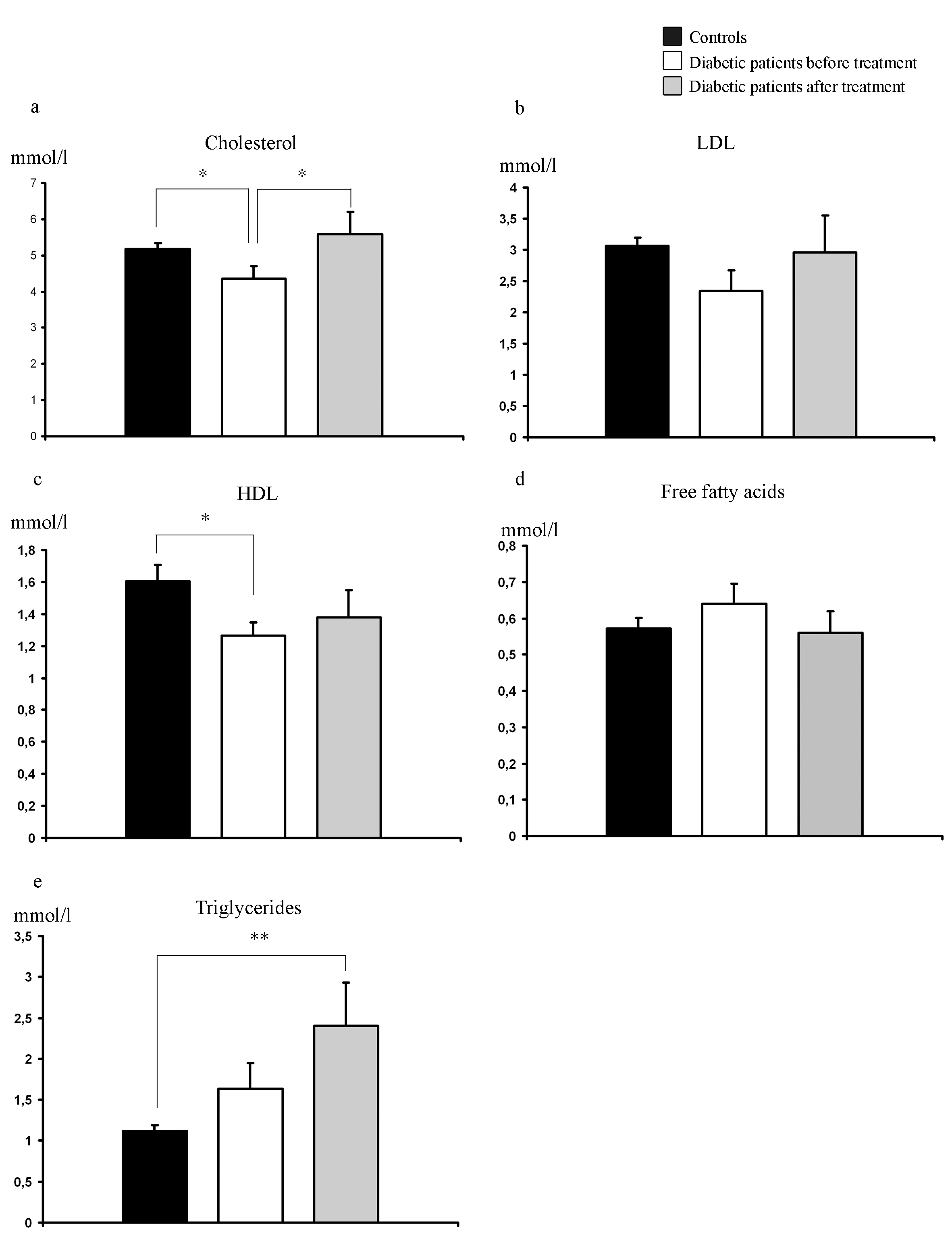

Supplement: Additional file 2 — Clinical variables II. The clinical variables cholesterol (a), LDL (b), HDL (c), free fatty acids (d) and triglycerides (e) of the healthy controls and diabetic patients before and after treatment with rosiglitazone for 90 days. Bars represent mean and error bars SEM. * = p < 0.05. [file 1758-5996-3-32-S2.JPEG]
